# Supplementary material for: The Role of Virulence Proteins in Protection Conferred by Bordetella pertussis Outer Membrane Vesicle Vaccines
Source: Vaccines (Basel). 2020 Jul 30;8(3):429. doi: 10.3390/vaccines8030429 (PMC7563335; doi:10.3390/vaccines8030429)
Supplement: Supplementary file 1 [file vaccines-08-00429-s001.pdf]

Supplemental Tables and Figures

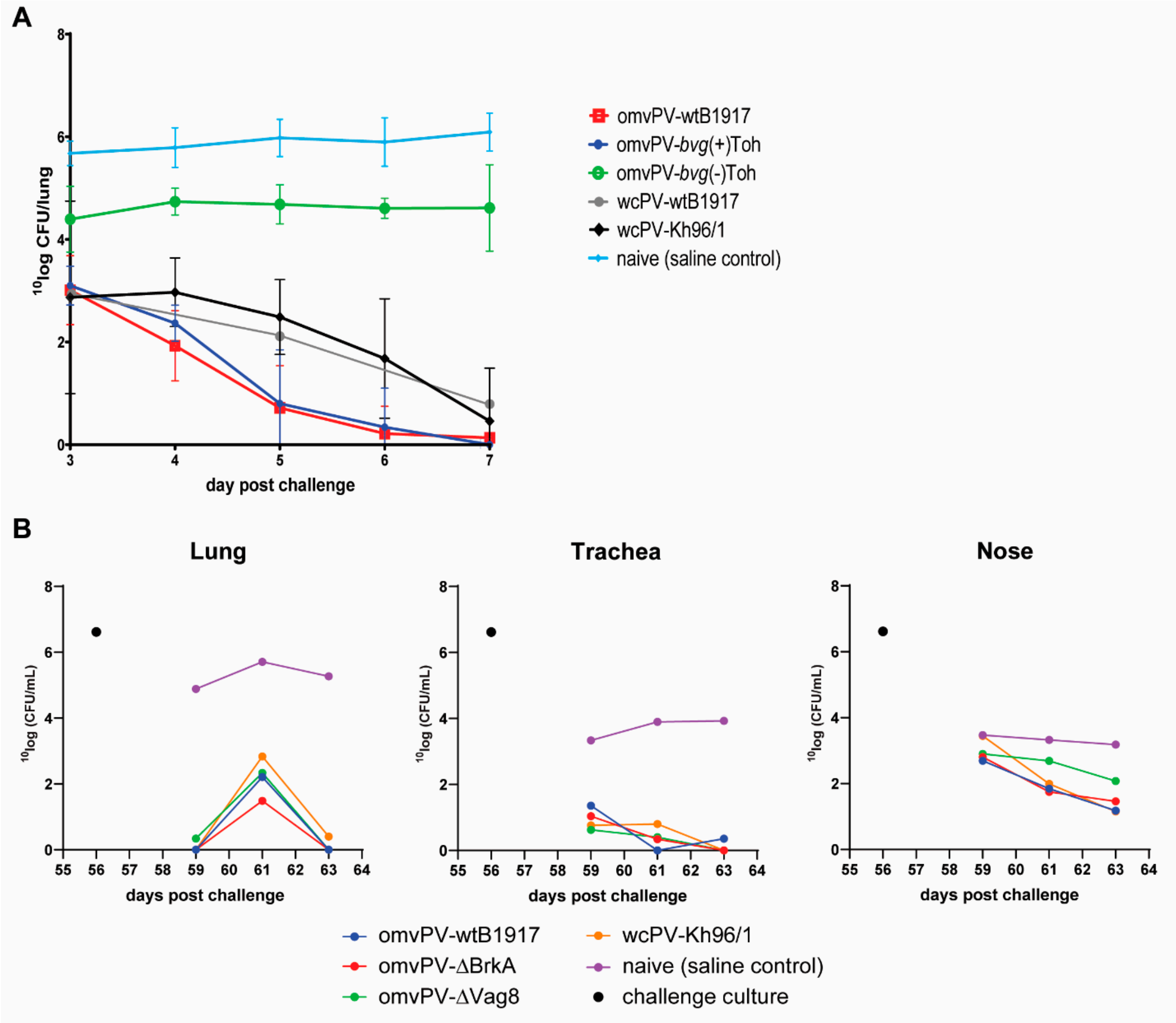

Supplemental Figure S1. Kinetics of bacterial clearance in respiratory tract. **(A)** Kinetics of CFU were determined in lungs of mice on 3, 4, 5, 6 and 7 days post challenge in groups immunized with omvPV-*bvg*(+)Toh, omvPV-*bvg*(+)Toh, omvPV-wtB1917, wcPV-Kh96/1 or saline (naive). Average and STDev are depicted. **(B)** Kinetics of colony forming units (CFU) were determined in in lung, trachea and nasal wash on 3, 5 and 7 days post challenge of mice immunized with omvPV-wtB1917, omvPV-ΔBrkA, omvPV-ΔVag8, wcPV-Kh96/1 or saline (naive). Average of data per vaccine group per day is shown. Black dot represents concentration of challenge culture prior to challenge.

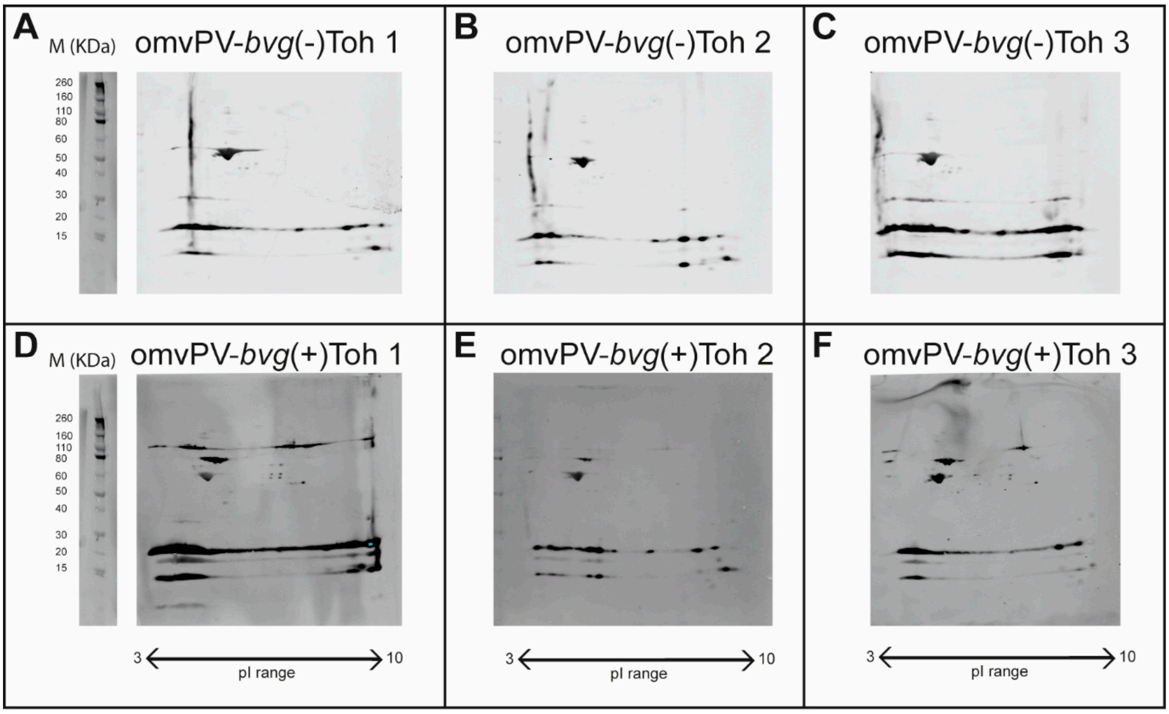

Supplemental Figure S2. Individual 2-Dimensional blots for immunoproteomic profiling omvPV-*bvg*(-)Toh and omvPV-*bvg*(+)Toh. Blots contain B1917 lysate (25μg) separated with 2D-electrophoresis on a isoelectric point range of 3 – 10 and molecular weight range of 260 – 3 kDa. Three individual blots per group were incubated with pooled serum from mice immunized with (**A-C**) omvPV-*bvg*(-)Toh or (**D-F**) omvPV-*bvg*(+)Toh in triplicate and spots visualized with an IR-800 labeled goat-anti-mouse IgG.

.

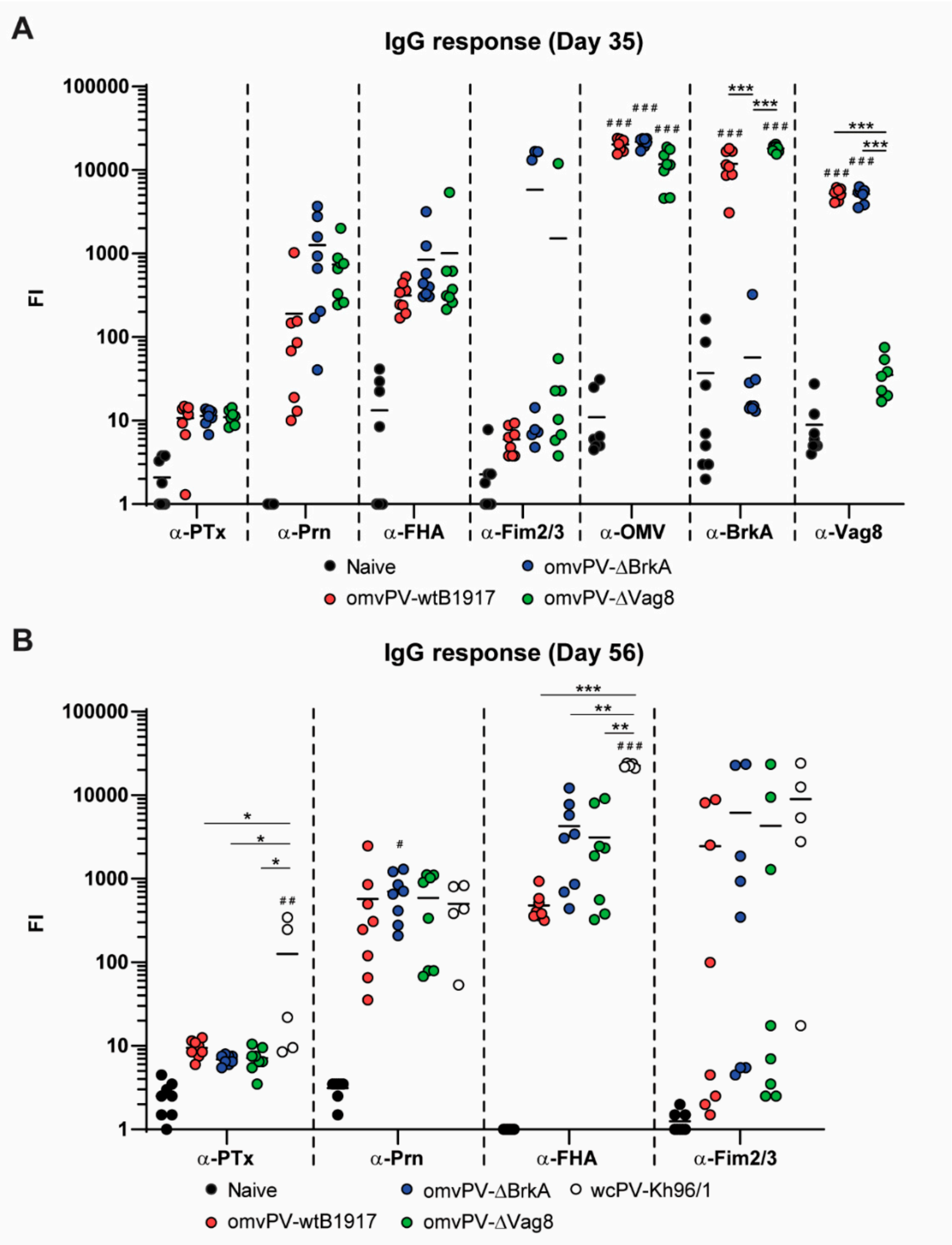

Supplemental Figure S3. Humoral responses on day 35 and day 56. On **A)** day 35, anti-Ptx, anti-Prn, anti-FHA, anti-Fim2/3, anti-OMV, anti-BrkA and anti-Vag8 IgG responses, **B)** day 56, anti-Ptx, anti-Prn, anti-FHA and anti-Fim2/3 IgG responses were determined in serum of naive mice or mice vaccinated with omvPV-wtB1917, omvPV-ΔBrkA, omvPV-ΔVag8 or wcPV-Kh96/1. Fluorescence intensity (FI) values are depicted on a [10]log scale axis. Significant differences compared to the naive mice are indicated as # $p \leq 0.05$, # # $p \leq 0.01$ and # # # $p \leq 0.001$ and actual P-values are shown in **Supplemental Table S2.** Significant differences between experimental groups is depicted with a line between both groups and * $p \leq 0.05$, ** $p \leq 0.01$ and *** $p \leq 0.001$.

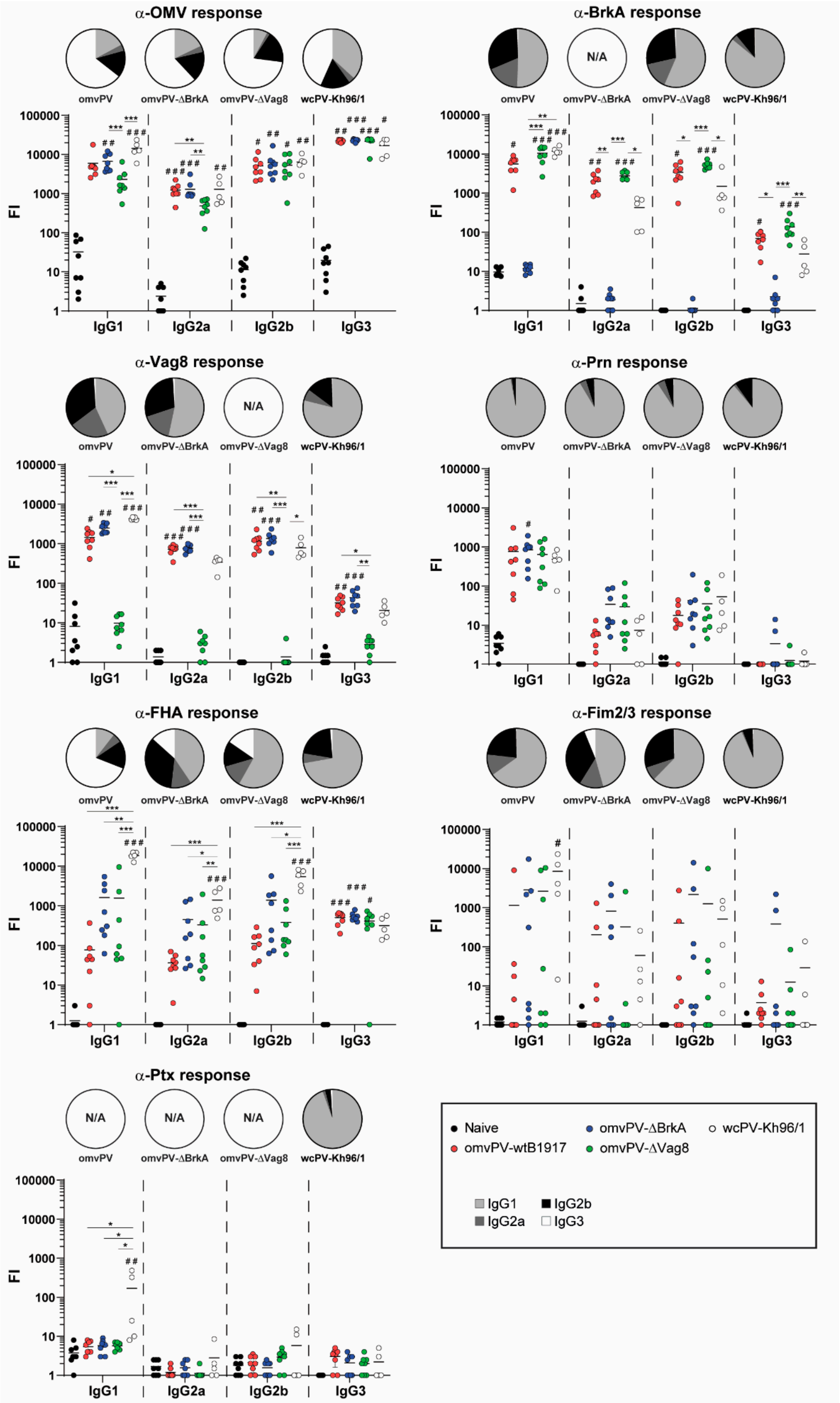
α-OMV response
α-BrkA response
α-Vag8 response
α-Prn response
α-FHA response
α-Fim2/3 response
α-Ptx response
omvPV
omvPV-ΔBrkA
omvPV-ΔVag8
wcPV-Kh96/1
N/A
FI
IgG1
IgG2a
IgG2b
IgG3
Naive
omvPV-wtB1917
omvPV-ΔBrkA
omvPV-ΔVag8
wcPV-Kh96/1
IgG1
IgG2a
IgG2b
IgG3

Supplemental Figure S4. Humoral subclass responses. Distribution of IgG subclass anti-OMV, anti-BrkA, anti-Vag8, anti-Prn, anti-FHA, anti-Fim2/3, anti-Ptx responses in naive mice or mice vaccinated with omvPV-wtB1917, omvPV-ΔBrkA, omvPV-ΔVag8 or wcPV-Kh96/1. Fluorescence intensity (FI) values are depicted on a ^{10}log scale axis. Significant differences compared to the naive mice are indicated as # $p \leq 0.05$, # # $p \leq 0.01$ and # # # $p \leq 0.001$ and actual P-values are shown in **Supplemental Table S2.** Significant differences between experimental groups is depicted with a line between both groups and * $p \leq 0.05$, ** $p \leq 0.01$ and *** $p \leq 0.001$. **.**

Supplemental Table S1. Raw data immunoproteomic profiling with 2-dimensional Western blotting (2DEWB).

Cluster	Protein ID	Accession number	mW (kDa)	pI	Coverage (%)	High confident identified peptides	gray value omvPV-*bvg*(-)Toh 1	gray value omvPV-*bvg*(-)Toh 2	gray value omvPV-*bvg*(-)Toh 3	Average gray value omvPV-*bvg*(-)Toh 1-3	gray value omvPV-*bvg*(+)Toh 1	gray value omvPV-*bvg*(+)Toh 2	gray value omvPV-*bvg*(+)Toh 3	Average gray value omvPV-*bvg*(+)Toh 1-3
**I**	CarB	Q7VY97	118.1	5.4	40	43	0.0617	0.0343	0.0667	0.0542	0.0059	0.0026	0.0185	0.0090
	AZ26-0755	A0A171K0C9	32	7.2	80	12	0.0101	0.0671	0.0352	0.0375	0.0055	0.0160	0.0084	0.0099
**II**	U1						1.0043	0.3678	1.6629	1.0117	0.0567	0.2296	0.0602	0.1155
	groL	P48210	57.4	5.2	86	60	0.4980	0.5649	0.6410	0.5679	0.1892	0.1237	0.3071	0.2067
**III**	groL 2	P48210	57.4	5.2	40	21	0.1199	0.0651	0.1496	0.1115	0.0505	0.0593	0.1027	0.0708
	U2						4.4664	2.6600	8.0628	5.0630	2.7591	7.0814	3.3281	4.3895
	U3						0.7152	0.8675	2.4633	1.3487	1.0047	2.9294	0.4898	1.4746
	U4						0.0002	0.0650	0.2914	0.1188	0.0117	0.1298	0.0878	0.0764
	odhB	Q7VZ17	41.8	5.5	75	20	0.0158	0.0164	0.0145	0.0156	0.0082	0.0112	0.0114	0.0102
	Tuf1	Q7TT91	41.8	5.5	78	24	0.0190	0.0117	0.0171	0.0160	0.0097	0.0262	0.0172	0.0177
**IV**	U5						0.1553	0.1423	0.2512	0.1829	0.3146	2.2625	0.3254	0.9675
	Vag8	Q79GN7	94.8	6.8	64	42	0.0119	0.0085	0.0276	0.0160	0.0326	0.6159	0.1126	0.2537
	U6						0.0200	0.0004	0.0152	0.0119	0.0220	0.1092	0.0278	0.0530
	rpsA	Q7VZG0	63.0	5.2	68	38	0.0345	0.0072	0.0051	0.0156	0.0280	0.0869	0.0413	0.0521
**V**	Brka	Q45340	103.3	7.1	47	30	0.0105	0.0080	0.0092	0.0092	0.0995	0.3554	0.2925	0.2491
	Brka 2	Q45340	103.3	7.1	29	17	0.0121	0.0058	0.0040	0.0073	0.0263	0.2899	0.1882	0.1681
	Brka 3	Q45340	103.3	7.1	29	16	0.0022	0.0081	0.0042	0.0048	0.0205	0.1543	0.1343	0.1030
	Prn	P14283	93.4	9.2	43	25	0.0107	0.0045	0.0043	0.0065	0.0158	0.0942	0.0359	0.0486
	U7						0.0018	0.0006	0.0003	0.0009	0.0160	0.0397	0.2239	0.0932
	bteA	A0A171JW44	68.6	5.3	88	37	0.0079	0.0030	0.0049	0.0052	0.0208	0.0510	0.0290	0.0336
	odhL	Q7VZ16	62.3	6.0	81	37	0.0083	0.0023	0.0057	0.0054	0.0210	0.1061	0.0210	0.0503
	dadA	Q7VXF6	45.9	7.4	47	14	0.0031	0.0068	0.0002	0.0033	0.0032	0.0173	0.0192	0.0132
	Ahcy	Q7VUL8	51.6	6.1	57	24	0.0105	0.0073	0.0078	0.0085	0.0170	0.0065	0.0274	0.0169
	aceF	Q7VZC4	57.2	5.9	60	24	0.0042	0.0037	0.0037	0.0037	0.0208	0.0086	0.0121	0.0139

Supplemental Table S2. Statistical significance of humoral responses.

**IgG total day 35**	**Ptx**	**Prn**	**FHA**	**Fim2/3**	**OMV**	**BrkA**	**Vag8**
Naive vs. omvPV-wtB1917	ns[1]	ns	ns	ns	***[2]	***	***
Naive vs. omvPV-ΔBrkA	ns	ns	ns	ns	***	Ns	***
Naive vs. omvPV-ΔVag8	ns	ns	ns	ns	***	***	ns
**IgG total day 56**	**Ptx**	**Prn**	**FHA**	**Fim2/3**	**OMV**	**BrkA**	**Vag8**
Naive vs. omvPV-wtB1917	ns	ns	ns	ns	***	*	*
Naive vs. omvPV-ΔBrkA	ns	*	ns	ns	***	ns	***
Naive vs. omvPV-ΔVag8	ns	ns	ns	ns	*	***	ns
Naive vs. wcPV-Kh96/1	**	ns	***	ns	*	*	**
**IgG1**	**Ptx**	**Prn**	**FHA**	**Fim2/3**	**OMV**	**BrkA**	**Vag8**
Naive vs. omvPV-wtB1917	ns	ns	ns	ns	ns	*	*
Naive vs. omvPV-ΔBrkA	ns	*	ns	ns	**	ns	**
Naive vs. omvPV-ΔVag8	ns	ns	ns	ns	ns	***	ns
Naive vs. wcPV-Kh96/1	**	ns	***	*	***	***	***
**IgG2a**	**Ptx**	**Prn**	**FHA**	**Fim2/3**	**OMV**	**BrkA**	**Vag8**
Naive vs. omvPV-wtB1917	N.A.[3]	ns	ns	ns	***	**	***
Naive vs. omvPV-ΔBrkA	N.A.	ns	ns	ns	***	ns	***
Naive vs. omvPV-ΔVag8	N.A.	ns	ns	ns	ns	***	ns
Naive vs. wcPV-Kh96/1	N.A.	ns	***	ns	**	ns	ns
**IgG2b**	**Ptx**	**Prn**	**FHA**	**Fim2/3**	**OMV**	**BrkA**	**Vag8**
Naive vs. omvPV-wtB1917	N.A.	ns	ns	ns	*	*	**
Naive vs. omvPV-ΔBrkA	N.A.	ns	ns	ns	**	ns	***
Naive vs. omvPV-ΔVag8	N.A.	ns	ns	ns	*	***	ns
Naive vs. wcPV-Kh96/1	N.A.	ns	***	ns	**	ns	ns
**IgG3**	**Ptx**	**Prn**	**FHA**	**Fim2/3**	**OMV**	**BrkA**	**Vag8**
Naive vs. omvPV-wtB1917	N.A.	N.A.	***	ns	**	*	**
Naive vs. omvPV-ΔBrkA	N.A.	N.A.	***	ns	***	ns	***
Naive vs. omvPV-ΔVag8	N.A.	N.A.	*	ns	***	***	ns
Naive vs. wcPV-Kh96/1	N.A.	N.A.	ns	ns	*	ns	ns

[1] ns = not significant; [2] * $p \leq 0.05$, ** $p \leq 0.01$ and *** $p \leq 0.001$; [3] N.A. = not applicable

Supplemental Table S3. Statistical significance of B-cell responses.

**Plasma B-cells (d35)**	**OMV**	**BrkA**	**Vag8**
Naive vs. omvPV-wtB1917	***[1]	**	***
Naive vs. omvPV-ΔBrkA	**	ns[2]	***
Naive vs. omvPV-ΔVag8	**	***	ns
**Memory B-cells (d56)**	**OMV**	**BrkA**	**Vag8**
Naive vs. omvPV-wtB1917	**	ns	ns
Naive vs. omvPV-ΔBrkA	**	ns	ns
Naive vs. omvPV-ΔVag8	ns	ns	ns
Naive vs. wcPV-Kh96/1	ns	ns	ns

[1] ** $p \leq 0.01$ and *** $p \leq 0.001$; [2] ns = not significant.

Supplemental Table S4. Statistical significance of T-helper responses.

**OMV stimulation**	**IFNγ**	**IL-10**	**IL-13**	**IL-17A**	**IL-4**	**IL-5**	**TNFα**
Naive vs. omvPV-wtB1917	**[1]	*	**	**	**	***	N.A.[2]
Naive vs. omvPV-ΔBrkA	**	***	***	**	**	*	N.A
Naive vs. omvPV-ΔVag8	***	**	**	**	***	ns	N.A
Naive vs. wcPV-Kh96/1	ns[3]	ns	ns	ns	ns	**	N.A
**BrkA stimulation**	**IFNγ**	**IL-10**	**IL-13**	**IL-17A**	**IL-4**	**IL-5**	**TNFα**
Naive vs. omvPV-wtB1917	**	ns	***	***	**	**	*
Naive vs. omvPV-ΔBrkA	N.A.	N.A.	N.A.	N.A.	N.A.	N.A.	N.A
Naive vs. omvPV-ΔVag8	***	***	***	***	***	***	***
Naive vs. wcPV-Kh96/1	ns	ns	ns	ns	ns	ns	**
**Vag8 stimulation**	**IFNγ**	**IL-10**	**IL-13**	**IL-17A**	**IL-4**	**IL-5**	**TNFα**
Naive vs. omvPV-wtB1917	***	*	***	**	***	**	*
Naive vs. omvPV-ΔBrkA	***	***	***	***	***	***	**
Naive vs. omvPV-ΔVag8	N.A	N.A	N.A	N.A	N.A.	N.A	N.A
Naive vs. wcPV-Kh96/1	ns	ns	ns	ns	ns	ns	**

[1] * $p \leq 0.05$, ** $p \leq 0.01$ and *** $p \leq 0.001$; [2] N.A. = not applicable; [3] ns = not significant.
